# Population structure of *Dugong dugon* across the Indo-Pacific revealed by historical mitogenomes

**DOI:** 10.1098/rsos.240599

**Published:** 2024-08-07

**Authors:** Lydia Hildebrand Furness, Oliver Kersten, Aurélie Boilard, Lucy Keith-Diagne, Cristina Brito, James H. Barrett, Andrew Kitchener, Richard Sabin, Shane Lavery, Stephanie Plön, Bastiaan Star

**Affiliations:** ^1^ Center for Ecological and Evolutionary Synthesis, Department of Biosciences, University of Oslo, Oslo 0313, Norway; ^2^ African Aquatic Conservation Fund, BP 80 Joal 23015, Senegal, West Africa; ^3^ NOVA University Lisbon, Lisbon 1099-085, Portugal; ^4^ Norwegian University of Science and Technology, Trondheim 7491, Norway; ^5^ Department of Natural Sciences, National Museums Scotland, Edinburgh EH1 1JF, UK; ^6^ School of Geosciences, University of Edinburgh, Edinburgh EH10 5HF, UK; ^7^ Vertebrates Division, Natural History Museum, London SW7 5BD, UK; ^8^ School of Biological Sciences, University of Auckland, Auckland 1010, New Zealand; ^9^ Department of Pathology, Stellenbosch University, Stellenbosch, South Africa; ^10^ Bayworld Centre for Research and Education, Port Elizabeth, 7602, South Africa

**Keywords:** ancient DNA, human exploitation, conservation genetics, population history, sirenians

## Abstract

*Sirenia*, an iconic marine taxon with a tropical and subtropical worldwide distribution, face an uncertain future. All species are designated ‘Vulnerable’ to extinction by the IUCN. Nonetheless, a comprehensive understanding of geographic structuring across the global range is lacking, impeding our ability to highlight particularly vulnerable populations for conservation priority. Here, we use ancient DNA to investigate dugong (*Dugong dugon*) population structure, analysing 56 mitogenomes from specimens comprising the known historical range. Our results reveal geographically structured and distinct monophyletic clades characterized by contrasting evolutionary histories. We observe deep-rooted and divergent lineages in the East (Indo-Pacific) and obtain new evidence for the relatively recent dispersal of dugongs into the western Indian Ocean. All populations are significantly differentiated from each other with western populations having approximately 10-fold lower levels of genetic variation than eastern Indo-Pacific populations. Additionally, we find a significant temporal loss of genetic diversity in western Indian Ocean dugongs since the mid-twentieth century, as well as a decline in population size beginning approximately 1000 years ago. Our results add to the growing body of evidence that dugong populations are becoming ever more susceptible to ongoing human action and global climate change.

## 1. Background

The field of ocean research is undergoing a paradigm shift with increased awareness of and compassion for species threatened by extinction due to human activity [1]. However, the significance of marine resource exploitation by past human societies remains poorly understood on a global scale [2]. This lack of knowledge especially applies to tropical and subtropical regions, which also comprise biodiversity hotspots at a disproportionately high risk from global climate change [3]. Moreover, these regions have been significant focal points of human hunting in the past with evidence of marine exploitation going back thousands of years [4]. In order to obtain a better understanding of global anthropogenic impacts [5], we require further investigation and focus on these tropical and subtropical marine ecosystems, and their key components.

The dugong (*Dugong dugon*) is an iconic, large, herbivorous marine mammal that inhabits tropical and subtropical shallow coastal regions and seagrass forests across the Indo-Pacific from East Africa to Vanuatu [6]. The dugong is the only surviving species of the once diverse and widespread Family *Dugongidae* [7], and is one of four extant sea cow (*Sirenia*) species. Their herbivory has important ecological consequences, exerting a significant top-down influence within seagrass meadows that is integral to ecosystem dynamics, productivity [8,9] and carbon sequestration [10,11]. At present, the dugong occurs in specific areas of the Indian Ocean, Red Sea, Persian Gulf and western Pacific Ocean [12]. The species has a long history of cultural and economic importance. Humans have settled near and used riverine and nearshore environments for millennia [13], and so it is unsurprising that the animals humans shared these environments with were both hunted and revered by Indigenous cultures [14]. For example, a long history of exploitation is evident from extensive archaeological bone mounds in northeastern Australia [15–17] and Neolithic sites on the Arabian Peninsula [18]. For many Indigenous peoples, the dugong was an important part of spiritual/magico-religious practice; their unusual shape and elusive behaviour gave them a cryptic and supernatural quality, with communities from the Torres Straits and Flores to Madagascar having complex hunting rites associated with their capture [17,19]. Nowadays, dugongs have become important for tourism, particularly at attractive dive sites in the Red Sea where divers can observe individuals in clear waters in their natural habitat [20].

The conservation status of the dugong is classified as ‘Vulnerable’ by the IUCN [6] with the primary population of East Africa classified as ‘Critically Endangered’ and one in New Caledonia as ‘Endangered’ [21,22]. The dugong is threatened by coastal development, illegal hunting, pollution, environmental degradation, entanglements and vessel collisions [23–26]. Their near exclusive dependence on tropical marine seagrasses [27,28] in coastal habitats makes them particularly vulnerable to both direct and indirect impacts of human activity. The dugongs’ range has been reduced by increasing population fragmentation due to ongoing human action. While post-colonial hunting rates from commercial industries are known to have been wholly unsustainable [29], the duration of such intensive exploitation remains unknown. Based on archaeological and historical evidence [30–32], it is probable that unsustainable practices had been established long before the modern era, and that the dugong may have been suffering prolonged human exploitation [14,16]. To date, the majority of dugong studies comprise localized population studies, focusing on specific seascapes and regionally distinct threats [33–39]. Localized extinctions in the South China Sea and Okinawa have been reported in recent years [40,41]. The main, substantial populations now persist in regions including Australia [42], the Persian Gulf [36] and New Caledonia [43]. The general population trend is one of decline, and the risk of extinction is particularly high in island groups [43]. Given these conservation concerns, we urgently need to broaden our understanding of dugong population structure and dispersal potential.

Despite the lack of obvious physical barriers, and the observed ability of some individuals to travel long distances (i.e. greater than 100 km) [44], mtDNA control region and microsatellite data show that distinct population clusters exist within the dugong range [12,45], and that populations have potentially greater genetic diversity in the Indo-Australian region [12,46]. In contrast, little geographic structuring has been detected among other populations, specifically in the western Indian Ocean [12]. A recent assessment of New Caledonian dugong population health, using the mtDNA control region, has revealed a genetically depauperate population, indicating that even some of the largest global dugong populations are now at significant risk of genomic degradation [43]. Nuclear whole genome data are limited to Australian waters, which still support the largest global population [47], and these data indicate a general decline in abundance since the last interglacial [48]. Our understanding of dugong population structure is therefore limited by the resolution of methods employed (e.g. [12]) or spatial scale, (e.g. [45] or [48]). As such, we are lacking a comprehensive population genetic assessment of the entire dugong range.

Recent advances in ancient DNA (aDNA) techniques have significantly altered our ability to obtain and analyse genomic data from historical and archaeological specimens [49] to be used in studying the biogeography of marine taxa [50–57] and conservation genomics [58]. Such advances have allowed the genomic analyses of historical museum specimens, which are of great relevance for species that are difficult or expensive to sample across their range.

In this study, we use established aDNA techniques to analyse the mitogenomes of 56 historical dugong individuals, from a sample set of 76 museum specimens, comprising the range of the species. Our main objective is for the improved resolution obtained by using entire mitogenomes to give a better insight into the population genetic structure and variation of the species. With these novel data, we expected to discover further geographic structuring that would elucidate prior findings, and possibly detect a loss of genetic diversity concurrent with archaeologically evidenced ancient hunting practices. Given the conservation concerns we have outlined, a comprehensive study such as this is urgently need to broaden our understanding of dugong population structure and dispersal potential in the context of ongoing environmental change.

## 2. Materials

### 2.1. Taxon sampling

**Figure 1.**
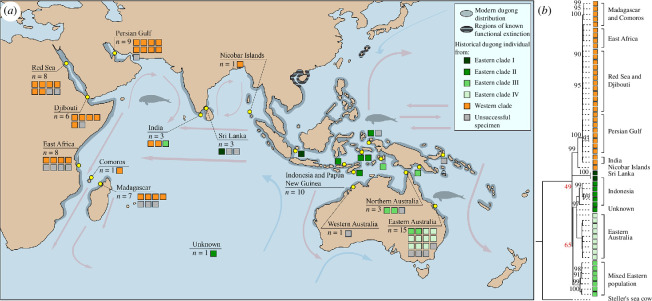
Distinct Indo-Pacific population structure of *D. dugon* revealed by historical mitogenomes. (*a*) Sample map of 56 dugong specimens (coloured boxes) showing the extant range of *D. dugon* (grey), including ranges of functional extinction (horizontal black lines). The specimens are coloured by the genetic clade (shades of green or orange) to which the individual was assigned. Arrows indicate major ocean currents, (*b*) Phylogenetic (ML) tree analyses of *D. dugon*, including a Steller’s sea cow specimen. Branches with bootstrap values >90 are labelled, and major clades with bootstrap values <90 are highlighted in red. Metadata for the broad locality of each specimen’s collection is given adjacent to the tree.

We sampled 76 individuals from known and major populations that span nearly all of the current and historical range of *D. dugon* (figure 1*a*). These individuals comprise 12 historical *D. dugon* bone specimens that were sampled *de novo* and 64 *D. dugon* DNA extracts that were used in a prior phylogeographic study targeting the mtDNA control region (D-loop) only (electronic supplementary material, table S1) [12]. We also sampled one archaeological Steller’s sea cow (*Hydrodamalis gigas*) bone which was used as an outgroup in downstream analyses. These samples came from a diverse set of historical museum collections (electronic supplementary material, tables S1 and S2). These specimens have been identified to species genetically or morphologically. Collection and/or accession dates were recorded for 67 total sampled individuals. Where collection date was unavailable, we used the accession date (see electronic supplementary material text).

### 2.2. Museum sampling

Bone samples were taken using a Dremel and diamond cutting blade. We took approximately 2 g bone pieces (see electronic supplementary material, text). Sampling for powder for the DNA extracts was conducted similarly by [12] and samples were shipped from museums to the University of Auckland, New Zealand, in accordance with CITES regulations. These samples were extracted—see [12] for details—for amplification of the mtDNA control region, and the extracts were frozen and stored. All bone samples and DNA extracts were transferred to the University of Oslo, Norway, in accordance with CITES regulations. Specifically, we used CITES permit exemption codes (exemption for scientific transfer) to ship from the University of Auckland, New Zealand (NZ010), and Naturalis Biodiversity Centre, The Netherlands (NL001), to Naturhistorisk Museum, University of Oslo, Norway (NO001).

## 3. Methods

### 3.1. Milling and extraction

All pre-PCR (polymerase chain reaction) protocols were performed in a clean lab at the University of Oslo, Norway, following strict aDNA precautions [59,60]. Bone samples were exposed to UV light before milling using a stainless steel mortar [61], where samples were crushed into a chunky powder. DNA extraction comprised a pre-digestion protocol [62] with modifications from [63], without the prior bleach wash (see electronic supplementary material, text for more details). Bone powder of weight 120–160 mg was used for each pre-digestion, which was then followed by an overnight digestion of 48 hours. Eluates were concentrated (Amicon−30 kDA centrifugal filter units) and the DNA collected using Minelute (Qiagen) columns according to the manufacturer’s instructions. DNA was eluted in 100 μl elution buffer (EB), preheated to 60°C [64]. A TE-Tween mix (1% TE buffer and Tween-20 mix) was added to the [12] DNA extracts at a volume of 1 μl per 20 μl of DNA extract to facilitate the release of DNA strands bound to the wall of the tube from long-term storage. A Qubit measurement was generated for all DNA extracts to optimize the dilution of ET SSB and P5 and P7 splinted adapters in the library build for the amount of input DNA, as recommended for the Santa Cruz reaction protocol [65].

### 3.2. Library, PCR and clean-up

Negative controls were included in extraction and library-build experiments. We generated libraries from a total of 76 historical dugong specimens, and one Steller’s sea cow. Extracted ancient and historical DNA was prepared for sequencing on the Illumina sequencing platform using the Santa Cruz reaction protocol, an approach that converts single-stranded and denatured double-stranded DNA into sequencing libraries in a single reaction [65]. Libraries were amplified using sample-specific P5 and P7 indexes. Amplification was initially carried out in triplicate 25 μl reactions and later adapted to single 75 μl reactions, as this made no difference to overall library complexity and sequencing results. Amplified libraries were cleaned and purified using AMPure® XP beads by Beckman-Coulter. All libraries were sequenced on an Illumina HiSeq 4000.

### 3.3. Mitogenomic analyses

Sequencing reads were processed with PALEOMIX [66] and mapping performed using BWA (*algorithm: mem, MinQuality*: 25). Reads were aligned to the *D. dugon* nuclear genome assembly and associated mitogenome ([67]—dnazoo.org) and the Florida manatee (*Trichechus manatus latirostris*) mitochondrial genome assembly (accession: PRJNA68243) [68], to identify any individuals that may have been misidentified by museums (see electronic supplementary material, text). Genome assemblies were downloaded from DNA Zoo ([67]—dnazoo.org [69]). BAM files for all sequenced historical specimens in this study have been released under the ENA accession number PRJEB74084. aDNA damage was investigated with mapDamage v. 2.2.1 [70] (electronic supplementary material, figure S1). Variant calling was performed using BCFtools v. 1.15.1 [71] with ploidy set to 1 (for haploid mitogenome data). Light VCF filtering was performed using BCFtools v. 1.15.1 [71] and VCFtools v. 0.1.16 [72] with the following parameters: *minQ* > 30.0*, min-meanDP* = 3*, remove indels* = yes [51]*, idepth* > 0.5. This produced a dataset of 56 mitogenomes with minimum of 0.5-fold coverage.

Principal component analysis (electronic supplementary material, figure S2) was conducted using PLINK v. 2.00a [73] and visualized in R Studio [74] using tidyverse v. 2.0.0 [75] and ggplot2 v. 3.4.4 [76]. The Steller’s sea cow sample (ID: SHG017) was used as an outgroup for phylogenetic and haplotype network analyses. Filtered VCFs were indexed and consensus sequences were built and compiled using BCFtools v. 1.15.1 *consensus* (*-H 1 a N -M N*) [71]. To visualize evolutionary relationships, IQ-TREE v. 2.2.2.3 [77] was used to generate a maximum likelihood (ML) tree using 1000 bootstrap replicates (*-m MFP -alrt 1000 -B 1000 AICc -bnni*), which was visualized and edited in FigTree v. 1.4.4 [78]. Tree confidence was assessed using bootstrap support values, and clades with branch values greater than 90 were considered strongly supported. An unrooted haplotype network was built using Fitchi [79] (with haploid used to specify mitochondrial (MT) data) and the required conversion from fasta to nexus file performed using ElConcatenero3 [80]. For further analyses, we used individuals with greater than 4.0-fold coverage (*idepth* > 4.0). Additionally, we assessed individual missingness (electronic supplementary material, figure S3) with VCFtools v. 0.1.16 [72], omitting any remaining individuals with considerable (*imiss* > 0.01) missing data. This subset of 41 individual mitogenomes (electronic supplementary material, table S4) was used for all subsequent analyses (see electronic supplementary material, text for details).

The multiple sequence alignment of our final subset (electronic supplementary material, table S4) was analysed in DnaSP v. 6 [81], where broad regional populations were specified according to museum location metadata and the data format edited for conversion to Arlequin file formats (*Genome = Haploid, Chomosome Location = Mitochondrial, Sites with alignment gaps = excluded*). Genetic distance between these populations was assessed using measures of absolute (*Dxy*) and relative (Φ_ST_) divergence, which were calculated in DnaSP v. 6 [81] and Arlequin [82], respectively. Φ_ST_ is a measure of population differentiation due to genetic structure, while *Dxy* assesses the genetic distance between populations based on sequence divergence. When analysed together Φ_ST_ and *Dxy* give a more nuanced understanding of population genetic structure and differentiation. In Arlequin, pairwise Φ_ST_ was calculated based on a pairwise distance matrix. *p*-values (electronic supplementary material, table S6) were generated in Arlequin to test the significance of pairwise Φ_ST_ using 1000 permutations. Genetic diversity was investigated on both spatial and temporal scales in DnaSP v. 6 [81], where we generated standard population genetic measurements under genetic differentiation and divergence analyses. Tajima’s D [83] and Fu and Li’s F [84]—statistics used in evolutionary biology to infer past population dynamics and detect selection—were generated for individual test groups within DnaSP v. 6 [81].

Female effective population size (*N_e_*) was modelled back in time for the species and for the eastern clades and western clade (as defined by the phylogeny) using a coalescent Bayesian skyline plot approach [85] implemented in BEAST v. 2.7.6 [86]. The multiple sequence alignment file was aligned using the MUSCLE alignment algorithm [87] in MEGA11 [88] and exported in nexus format. Jmodeltest2 v. 2.1.10 [89] was used to determine the best nucleotide model; best fitting models were determined using ΔBIC (HKY+G). In BEAUti [86], the file was annotated: *site model = HKY*; *gamma category count* = 4; *strict clock*; *normal distribution*; *upper/lower bound rates:* 7.0 × 10^−9^–7.5 × 10^−8^ *substitutions*/*site*/*year* (based on *Odobenus rosmarus*, see [51]). The output was then run in BEAST v. 2.7.6 [86] (10 000 000 iterations; 10% burn-in; logged every 10 000). Log and tree files were read into Tracer v. 1.7.2 [90] confirming convergence (ESS > 200) after which a coalescent Bayesian skyline plot was generated.

## 4. Results

### 4.1. DNA yield and library success

We obtained approximately 3.25 billion paired total sequencing reads for 66 dugong specimens from across the Indo-Pacific region (figure 1*a*). Specimens yielded between 0.06 and 65% endogenous DNA, with 3–54% mitochondrial clonality and an average read length of 88.4 bp (electronic supplementary material, table S3). Sequencing reads from all historical specimens show the typical fragmentation and deamination patterns expected with post-mortem DNA degradation, with recent specimens (i.e. from 1995) showing little post-mortem sequence modification (electronic supplementary material, figure S1). Of the 66 dugong libraries that were successfully amplified and sequenced, 56 passed our initial filtering with 0.5×-fold coverage of the mitogenome.

### 4.2. Population structure and phylogenetic analyses

Principal component analysis of the 56 mitogenomes yielded three distinct clusters (electronic supplementary material, figure S2). An ML phylogenetic analysis shows that these three clusters can be broadly resolved into five major monophyletic clades. These clades are largely geographically restricted; they occur on either side of the Indo-Pacific range apart from a limited central zone off the Indian subcontinent where they co-occur (figure 1).

**Figure 2.**
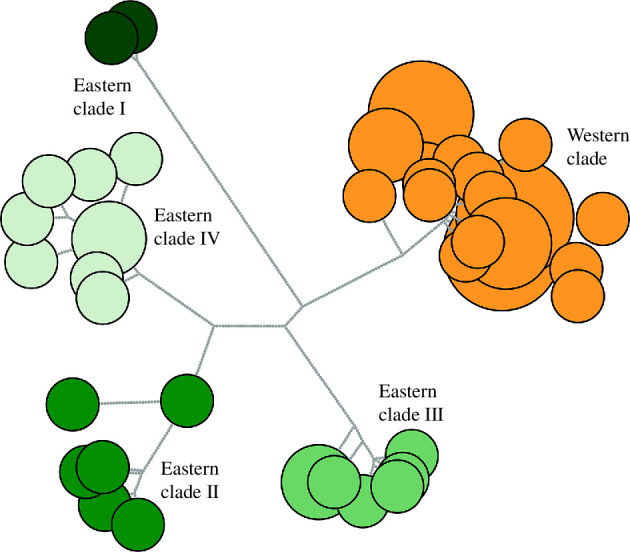
An unrooted haplotype network of 56 dugong individuals reflecting vastly different levels of divergence between eastern and western clades. The network nodes are coloured by the clade to which the individual mitogenomes were assigned genetically. Node size corresponds to number of individuals sharing that haplotype. Grey circles are indicative of single nucleotide polymorphisms (SNPs).

The phylogeny (figure 1*b*, see also electronic supplementary material, figure S4 for greater detail) and haplotype network (figure 2) of the 56 historical dugong samples reveal significant population structure across the Indo-Pacific range. The western clade (orange, figures 1 and 2) includes mitogenomes from eight broad regions across the western, and parts of the northern, Indian Ocean. In the eastern Indo-Pacific, there are at least three well-supported clades (II, III, IV; shades of green, figures 1 and 2). Each clade is supported with bootstrap support values >90. The exact relationships between these clades are less well defined, with a bootstrap support value of 65. We also observe a genetically distinct clade (I) in this region composed of two individuals (dark green, figures 1 and 2). The relationship of this clade with eastern clades II, III, IV, within the major east/west bifurcation, is less certain, with a bootstrap support value of 49 (see electronic supplementary material, text). Within the clades, subclades in the phylogeny are largely geographically structured. Many regionally defined clusters are supported with bootstrap support values >90, including those in the western clade such as Madagascar/Comoros (figure 1*b*).

The unrooted haplotype network (figure 2) has a total of 409 variable sites (including missing data given that the programme cannot discriminate) across the 56 mitogenomes. We observe lower levels of unique haplotypes among individuals from the western clade (with localities including the Persian Gulf, Red Sea and Djibouti) than those from the eastern clades (with localities including Indonesia and Australia; for figure 2 coloured according to museum location metadata, see electronic supplementary material, figure S5). Individuals in the eastern clades are separated by greater numbers of single nucleotide polymorphisms (SNPs) compared with their western clade conspecifics.

### 4.3. Genomic variation and population diversification

Nucleotide diversity (*π*) estimates within *D. dugon* are based on a subset of 41 mitogenomes with sufficiently high coverage to reduce the impact of missing data (electronic supplementary material, table S4). This dataset includes 257 segregating sites (*S*) leading to a *π* of 0.0030 (table 1). We detect 31 unique haplotypes, six of which are shared by multiple individuals. Haplotype sharing occurs almost exclusively in the western clade (five out of six instances).

**Table 1.** Genetic diversity of *D. dugon* within six broad and regionally defined populations, and between eastern and western genetic clades. Standard measures given are the number of individuals (*N*); haplotype diversity (*h*); the number of haplotypes (*Nh*); segregating sites (*S*); nucleotide diversity (*π*); Tajima’s D (TD); Fu and Li’s F (*F*). Significant *p*-values (<0.05) in *TD* and *F* analyses are indicated with an asterisk. ID indicates insufficient individuals to compute the statistic.

population	code	*N*	*h*	*Nh*	*S*	*π*	TD	*F*
Australia	AUS	9	0.97	8	96	0.0027	1.343	2.082
Indonesia and Papua New Guinea	IPG	6	1.00	6	150	0.0036	−0.762	1.707
India, Sri Lanka and Nicobar Islands	ISN	3	1.00	3	109	0.0046	ID	ID
Persian Gulf	PER	5	0.80	3	2	0.0001	1.459	−0.186
Red Sea and Djibouti	RSD	10	0.80	5	9	0.0002	−0.139	1.072
East Africa, Madagascar and Comoros	EMC	8	0.89	6	15	0.0003	−0.261	−1.580
total		41	0.98	31	257	0.0030	−0.745	−0.335
eastern clades		16	0.99	15	202	0.0038	0.0843	0.353
western clade		25	0.95	16	49	0.0004	−1.8433*	−3.217*

Between regionally defined populations (table 1), *π* ranges from 0.0001 to 0.0046, a 46-fold difference between the highest (ISN) and lowest (PER) populations measured. Haplotype diversity (*h*) and total *S* are significantly greater in populations in the eastern than the western Indo-Pacific region and significant variation in the spatial distribution of genetic variation (*χ^2^ p‐*value *=* 0.0019**) is apparent across dugong populations. Neutrality tests show no significant values for Tajima’s D (TD) and *F* statistics in any populations.

Of the 257 variable sites, 193 sites are polymorphic in the eastern clades and monomorphic in the western clade, while 40 sites are polymorphic in the western clade and monomorphic in the eastern clades (figure 1*b*; see electronic supplementary material, tables S4 and S5). Overall, only nine sites were polymorphic in both the east and west. Nucleotide diversity (*π*) in the western clade (*n* = 25) is 0.0004, and 0.0038 in the eastern clades (*n* = 16). The difference between average number of nucleotide differences (*k*) is approximately 10-fold between east (*k =* 60.392) and west (*k =* 6.887). Although *π* is approximately 10-fold higher in the eastern clades (table 1), overall genetic differentiation between clades did not reach significance (*χ* ^2^ *p*‐value = 0.0869 ns). Neutrality tests show significantly negative values (*p*‐value ≤ 0.05) for TD and *F* statistics within the western clade (table 1).

**Figure 3.**
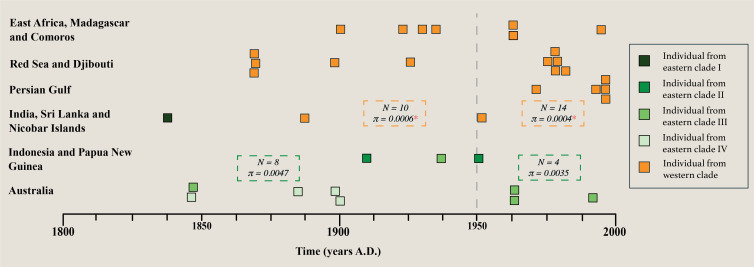
Temporal genomics of *D. dugon* in the Indo-Pacific region demonstrate that there has been a significant loss of genetic diversity in the western clade over recent times. A timeline from 1800 to 2000 AD shows the temporal distribution of individuals used to measure the loss of genetic diversity over time. Data used are museum collection and accession dates (see electronic supplementary material, table S6). The dashed line at 1950 visually divides the two temporal periods tested for each clade following Plön *et al.* [12]. Individuals are coloured by clade. A number of individuals (*n*) and nucleotide diversity (*π*) for each tested clade subset are given within the dashed boxes, and a significant loss of diversity (*χ* ^2^ *p*‐value ≤ 0.05) is indicated with a red asterisk.

The potential for loss of diversity over time was tested for individuals with >4-fold coverage and for which we had a collection or accession date (*n =* 36) (see electronic supplementary material, table S6 and text). We observe no significant difference in genetic variation in the eastern clades before (*n* = 8) and since (*n* = 4) 1950 (*χ* ^2^ *p*‐value = 0.2851 ns), although a decrease in nucleotide diversity (*π*) from 0.0047 to 0.0035 is apparent (figure 3). Nevertheless, we do identify a significant loss of genetic diversity in the western clade before (*n* = 10) and since (*n* = 14) 1950 (*χ* ^2^ *p*‐value = 0.0458*). Neutrality tests show no significant values for TD and *F* statistics within these subsets (electronic supplementary material, table S7).

**Figure 4.**
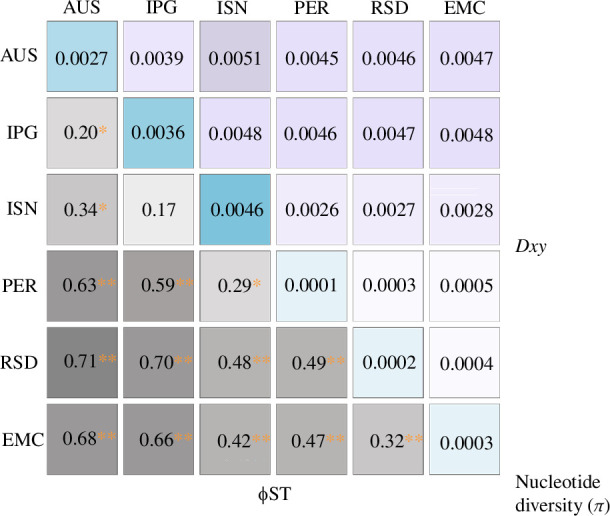
Significant population structure between regionally defined dugong populations in the Indo-Pacific based on MT genome data. A heatmap depicts pairwise measures of absolute (*Dxy*: pink) and relative (Φ_ST_: grey) divergence between populations. Nucleotide diversity (*π*: blue) within populations is given on the diagonal. Populations are coded as shown in table 1. Darker colours denote higher divergence or greater diversity. Significant *p*-values are indicated with a red asterisk (*≤0.05; **≤0.01).

Substantial population diversification is observable between broad-scale regions based on pairwise measures of absolute and relative divergence, as well as intrapopulation genetic diversity and nucleotide diversity (π) (figure 4). Levels of π were the greatest within eastern Indian Ocean populations, specifically ISN and IPG. Pairwise Φ_ST_ values show significant (*p*‐value ≤ 0.01) divergence between almost all population pairs (electronic supplementary material, table S8). Measures of pairwise Φ_ST_ are concordant with the geographical proximity of populations, suggesting that the majority of structuring may be explained on a spatial scale.

*Dxy* (absolute divergence) measurements are high within east–east and east–west pairwise tests (0.0051–0.0026), along with high pairwise Φ_ST_ (0.20–0.71). One exception to this is IPG/ISN, which does not show siginificant differentiation (*p*‐value ≥ 0.05); here we observe high *Dxy* (0.0048) and relatively low Φ_ST_ (0.17). By contrast, west–west pairwise tests have high Φ_ST_ (0.32–0.49) and low *Dxy* (0.0003–0.0005).

### 4.4. Long-term population dynamics

**Figure 5.**
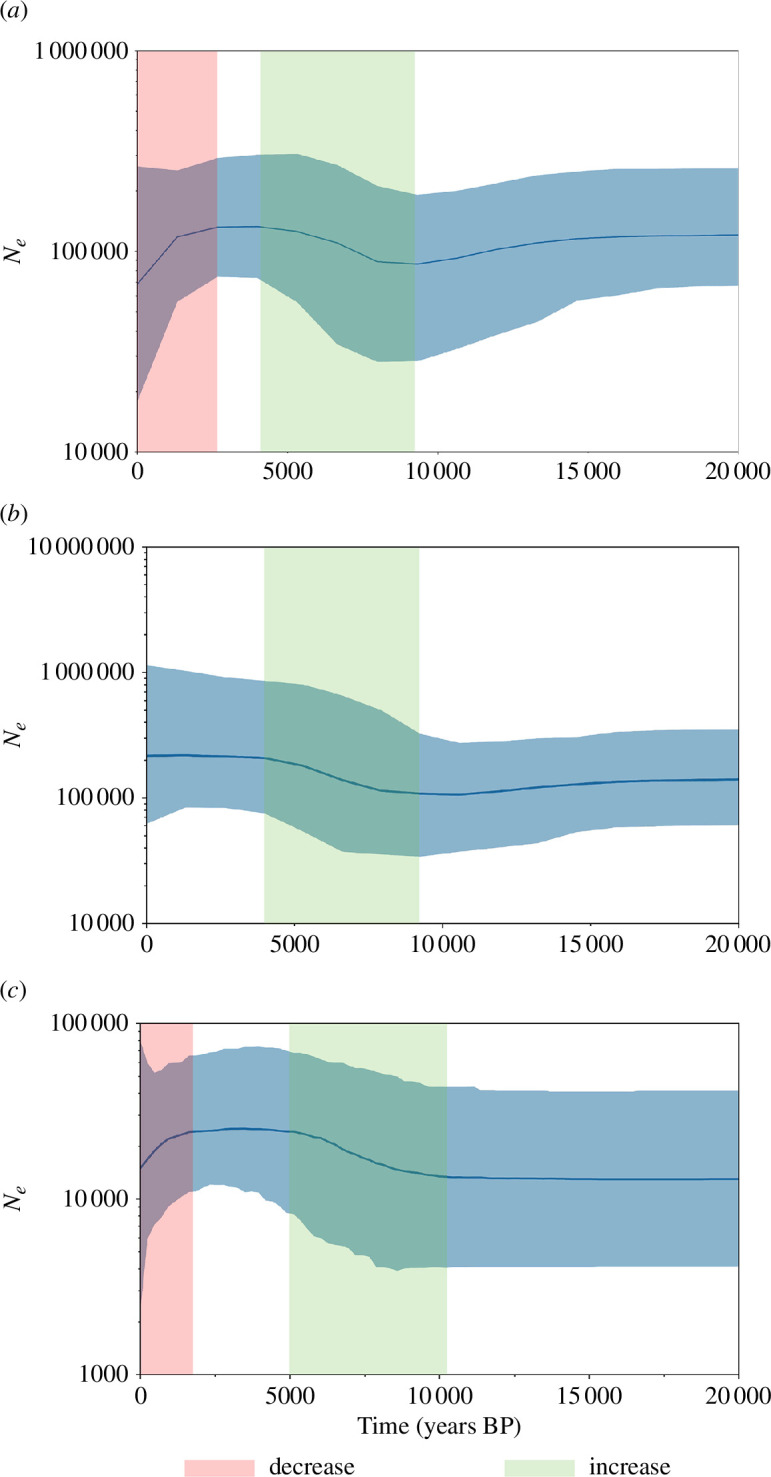
Long-term demography of *D. dugon* investigated using coalescent Bayesian skyline plots shows recent rapid reduction of female effective population size in western Indo-Pacific populations. These plots show female effective population size (*N*_e_) of (*a*) all *D. dugon* individuals, (*b*) the eastern clades’ individuals and (*c*) the western clade individuals, based on an assumed rate of 7.0 × 10^−9^–7.5 × 10^−8^ substitutions/site/year. Time is given in thousands of years ago (kya). Colour bands indicate increasing (green) and decreasing (red) *N*_e_.

The demographic history of *D. dugon* was investigated using coalescent Bayesian skyline plots and time estimates obtained based on a standard substitution rate of 7.0 × 10^−9^–7.5 × 10^−8^ substitutions/site/year (figure 5). Overall, female *N*_*e*_ for *D. dugon* experienced two periods of reduction in recent times (figure 5*a*). This appears to be due to a slight reduction in *N*_e_ in the east approximately 12 500 years ago (figure 5*b*), and a more dramatic reduction in the west over the last 1000 years (figure 5*c*). Notably, *N*_e_ in the east (figure 5*b*) has consistently been approximately 10-fold greater than that in the west (figure 5*c*).

## 5. Discussion

In this study, we used aDNA from historical specimens to improve our understanding of the population structure and temporal genomics of *D. dugon.* We observe significant population differentiation across the Indo-Pacific, associated with distinct evolutionary lineages. We find that levels of dugong genetic diversity are approximately 10-fold higher in the eastern compared with the western Indo-Pacific region. A significant decline in female *N*_e_ over the last millennium was observed in the West. We discuss the implications of our findings in the following text.

First, we find that the Indo-Pacific population divergence of *D. dugon* is largely associated with evolutionarily distinct clades that are largely geographically restricted to either the western or eastern parts of the range. Significant population structure is also found within these regions. For instance, in a previous study based only on mtDNA D-loop data, Madagascar/Comoros was the only western Indian Ocean population to be distinguished genetically [12]. Based on the higher resolution of our mitogenomic data, we find here that all of our sampled populations from this region (PER, RSD, EMC) are in fact significantly different (Φ_ST_) from one another. Moreover, the observed patterns of high Φ_ST_ and low *Dxy* among these populations suggest a more recent divergence than among the eastern clades. The data show a low sequence divergence, despite populations being significantly differentiated; absolute divergence tends to become high later relative to relative divergence because it reflects the accumulated changes in sequences over time. In conjunction with significantly negative TD and *F* values for the western clade, which are indicative of recent population expansion or a bottleneck [83,84,91], our findings point towards a relatively recent dispersal from a common ancestor of all modern western Indian Ocean dugongs across the Indo-Pacific.

Eastern clades, by contrast, comprised specimens with considerably deeper evolutionary divergence, and pairwise population divergence was driven by strong phylogeographic structure, with both high Φ_ST_ and *Dxy*. Additionally, we found evidence of a genetically distinct clade (I) concordant with recent findings of potentially long-isolated and genetically distinct dugong aggregations in nearby Andaman seascapes [46,92]. Overall, the divergence patterns observed are consistent with the hypothesis that diversification and dispersal of *D. dugon* initially took place in the east, with some dispersal as far as South Asia, resulting in deeply rooted and diverse clades. The current western Indian Ocean clade resulted from a relatively recent colonization event. The biographical causes behind the current patterns of genetic variation are difficult to assess from these data alone; a greater sample size and use of whole genome sequencing may allow us to detect more detailed population structuring—specifically the placement of the deeper clades of which the branching could not be confidently resolved—as well as to better assess our hypothesis of a relatively recent dispersal of the modern western Indian Ocean dugong. Further, it should be considered that this dispersal could be linked to past environmental change, such as the disappearance of Sundaland after the Last Glacial Maximum [93] and/or the spread of seagrass meadows, which could have allowed dugongs to spread out across the Indo-Pacific.

Second, we determined that female *N*_e_ has been relatively stable over the last 20 000 years and that reductions in *N*_e_ have only occurred during the last millennium. This observation is in contrast to archaeological evidence, specifically the extensive dugong bone mounds excavated in the Arabian Peninsula, which spawned the hypothesis that dugong hunting has been intensive, and possibly unsustainable, since the Neolithic [14,16]. We did not observe the expected mitogenomic consequences, i.e. a loss of genetic diversity (e.g.[94,95]) that would support this. It is possible that the way in which people were hunting in the distant past could explain this finding. For example, if ancient hunting was on a local scale with heavy exploitation limited to populations that could be easily reached, the extirpated populations may not be represented in this historical dataset or the cumulative effect of such hunting practices on the entire global population may not be enough to leave a mitogenomic signal. It is possible that additional analyses (i.e. targeting the nuclear genome), could detect such an effect. Additionally, if hunting was sex-biased, i.e. males are targeted, a mitogenome study such as this would again not detect the expected genomic consequences of heavy exploitation. Finally, the recent reduction in *N*_e_ could be due to population structure [96]. Nonetheless, we find this reduction in the western populations which have considerably lower population structure compared with the eastern populations. We, therefore, consider it unlikely that such a structure drives the reduction of *N*_e_ in western populations.

Third, mitogenomic variation differs greatly across dugong populations, with considerably lower levels of genetic diversity in the west than that in the east. Of particular concern is that one of the largest global dugong stocks, the Persian Gulf population [36], shows the lowest genetic diversity (*π* = 0.001). This finding is similar to recent findings in New Caledonia of another of the largest global stocks comprising only three distinct haplotypes and having extremely low nucleotide diversity [43]. While we emphasize the urgent need for conservation management of the dugong, our findings demonstrate that additional conservation priority should be given to western populations who could be at disproportionate risk due to their considerably lower levels of genetic diversity, a limiting factor for adaptive potential, and relatively low population size. Nevertheless, with further anthropogenically induced extinctions [40,41], the higher level of unique genetic diversity in the eastern clades, and therefore, the uniqueness of the species remains under considerable threat. Overall, the distinct population structuring found throughout the Indo-Pacific adds to the existing evidence [97] for assigning conservation management units.

## 6. Conclusion

Our dataset represents the largest range-wide historical mitogenome study of *D. dugon* to date. The findings provide considerably greater resolution of species-wide genetic diversity and population structure across the Indo-Pacific. While prior studies had uncovered potentially divergent lineages of the dugong, our findings are biologically significant as a distinct split across the Indo-Pacific had not yet been reported and investigated at this level of detail. We have demonstrated that dugong female effective population size has been declining and that there has been a measurable loss of genetic diversity in the western portion of their range in recent history. Overall, our data add to the growing body of evidence that global dugong populations are significantly fragmented and becoming increasingly less genetically diverse, making them ever more susceptible to ongoing anthropogenic threats and global climate change.

## Data Availability

All raw sequence data has been released in the appropriate repositories. The BAM files for all historical specimens are released under the ENA accession no. PRJEB74084. Supplementary material is available online [98].

## References

[1] Stenseth NC *et al* . 2020 Attuning to a changing ocean. Proc. Natl Acad. Sci. USA **117** , 20363–20371. (10.1073/pnas.1915352117) 32817527 PMC7456143

[2] Holm P , Hayes P , Nicholls J . 2024 Historical marine footprint for Atlantic Europe, 1500–2019. Ambio **53** , 624–636. (10.1007/s13280-023-01939-9) 38281258 PMC10920564

[3] Brown SC , Mellin C , García Molinos J , Lorenzen ED , Fordham DA . 2022 Faster ocean warming threatens richest areas of marine biodiversity. Glob. Chang. Biol. **28** , 5849–5858. (10.1111/gcb.16328) 35795987 PMC9544294

[4] O’Connor S , Kealy S , Reepmeyer C , Samper Carro SC , Shipton C . 2022 Terminal Pleistocene emergence of maritime interaction networks across Wallacea. World Archaeol. **54** , 244–263. (10.1080/00438243.2023.2172072)

[5] Santana-Cordero AM , Szabó P , Bürgi M , Armstrong CG . 2024 The practice of historical ecology: what, when, where, how and what for. Ambio **53** , 664–677. (10.1007/s13280-024-01981-1) 38441861 PMC10992833

[6] Marsh H , Sobtzick S . 2019 Dugong dugon (amended version of 2015 assessment). The IUCN red list of threatened species 2019: e.T6909A160756767. See 10.2305/IUCN.UK.2015-4.RLTS.T6909A160756767.en.

[7] Domning DP . 2018 Sirenian evolution. In Encyclopedia of marine mammals (eds B Würsig , JGM Thewissen , KM Kovacs ), pp. 856–859, 3rd edn. San Diego, CA, USA: Academic press. (10.1016/B978-0-12-804327-1.00229-6)

[8] Bullen CD . 2020 A marine megafaunal extinction and its consequences for kelp forests of the North Pacific. Thesis, University of British Columbia, Vancouver, Canada.

[9] Estes JA , Burdin A , Doak DF . 2016 Sea Otters, kelp forests, and the extinction of Steller’s sea cow. Proc. Natl Acad. Sci. USA **113** , 880–885. (10.1073/pnas.1502552112) 26504217 PMC4743786

[10] Romero J , Pérez M , Mateo MA , Sala E . 1994 The belowground organs of the mediterranean seagrass Posidonia oceanica as a biogeochemical sink. Aquat. Bot. **47** , 13–19. (10.1016/0304-3770(94)90044-2)

[11] Russell BD , Connell SD , Uthicke S , Muehllehner N , Fabricius KE , Hall-Spencer JM . 2013 Future seagrass beds: can increased productivity lead to increased carbon storage. Mar. Pollut. Bull. **73** , 463–469. (10.1016/j.marpolbul.2013.01.031) 23453889

[12] Plön S , Thakur V , Parr L , Lavery SD . 2019 Phylogeography of the dugong (Dugong dugon) based on historical samples identifies vulnerable Indian Ocean populations. PLoS One **14** , e0219350. (10.1371/journal.pone.0219350) 31509531 PMC6738584

[13] Bailey G . 2004 World prehistory from the margins: the role of coastlines in human evolution. J. Interdiscip. Stud. Hist. Archaeol. **1** .

[14] Ponnampalam LS , Keith-Diagne L , Marmontel M , Marshall CD , Reep RL , Powell J *et al* . 2022 Historical and current interactions with humans. In Ethology and behavioral ecology of Sirenia (ed. H Marsh ), pp. 299–349. Cham, Switzerland: Springer International Publishing.

[15] Minnegal M . 1984 Dugong bones from Princess Charlotte Bay. Aust. Archaeol. **18** , 63–71. (10.1080/03122417.1984.12092932)

[16] McNiven IJ , Bedingfield AC . 2008 Past and present marine mammal hunting rates and abundances: dugong (Dugong dugon) evidence from Dabangai Bone Mound, Torres Strait. J. Archaeol. Sci. **35** , 505–515. (10.1016/j.jas.2007.05.006)

[17] McNiven IJ , Feldman R . 2003 Ritually orchestrated seascapes: hunting magic and dugong bone mounds in Torres Strait, NE Australia. C.A.J. **13** , 169–194. (10.1017/S0959774303000118)

[18] Méry S , Charpentier V , Auxiette G , Pelle E . 2009 A dugong bone mound: the Neolithic ritual site on Akab in Umm al-Quwain, United Arab Emirates. Antiquity **83** , 696–708. (10.1017/S0003598X00098926)

[19] Forth G . 2021 Rare animals as cryptids and supernaturals: the case of dugongs on Flores Island. Anthrozoös. **34** , 61–76. (10.1080/08927936.2021.1878681)

[20] Nasr D , Shawky AM , Vine P . 2019 Status of red sea dugongs. In Oceanographic and biological aspects of the Red Sea (eds NMA Rasul , ICF Stewart ), pp. 327–354. Cham, Switzerland: Springer International Publishing. (10.1007/978-3-319-99417-8)

[21] Trotzuk E *et al* . Dugong dugon (east african coastal subpopulation). Dugong. IUCN Red List Cat. & Crit. **2022** , 1–19. (10.2305/IUCN.UK.2022-2.RLTS.T218582764A218589142.en)

[22] Trotzuk E , Findlay K , Taju A , Cockcroft V , Guissamulo A , Araman A , Matos L , Gaylard A . 2022 Focused and inclusive actions could ensure the persistence of East Africa’s last known viable dugong subpopulation. Conservat. Sci. Prac. **4** , e12702. (10.1111/csp2.12702)

[23] Borsa P . 2006 Marine mammal strandings in the New Caledonia region, Southwest Pacific. C. R. Biol. **329** , 277–288. (10.1016/j.crvi.2006.01.004) 16644500

[24] Das HS , Dey SC . 1999 Observations on the dugong, dugong dugon (muller), in the andaman and nicobar islands, india. J. Bombay Nat. Hist. Soc. **96** , 195–198. https://biostor.org/reference/151781

[25] Raghunathan C , Venkataraman K , Rajan PT . 2012 Status of sea cow, dugong (Dugong dugon) in Andaman and Nicobar Islands. Nat. Environ. Pollut. Technol. **11** , 105–112.

[26] Schoeman RP , Patterson-Abrolat C , Plön S . 2020 A global review of vessel collisions with marine animals. Front. Mar. Sci. **7** , 292. (10.3389/fmars.2020.00292)

[27] Domning DP . 1981 Sea cows and sea grasses. Paleobiology **7** , 417–420. (10.1017/S009483730002546X)

[28] Marsh H , Grech A , McMahon K . 2018 Dugongs: seagrass community specialists. In Seagrasses of Australia: structure, ecology and conservation (eds AWD Larkum , GA Kendrick , PJ Ralph ), pp. 629–661. Cham, Switzerland: Springer International Publishing. (10.1007/978-3-319-71354-0)

[29] Marsh H . 2009 Dugong: *Dugong dugon* . In Encyclopedia of marine mammals (eds WF Perrin , B Würsig , JGM Thewissen ), pp. 332–335, 2nd edn. London, UK: Academic Press. (10.1016/B978-0-12-373553-9.00080-8)

[30] Cansdale GS . 1970 All the animals of the Bible lands. Grand Rapids, MI: Zondervan Publishing House.

[31] Beech M . 2010 Mermaids of the arabian gulf: archaeological evidence for the exploitation of dugongs from prehistory to the present. Liwa. **2** , 3–18. https://www.researchgate.net/publication/269105636

[32] Crouch J , McNiven IJ , David B , Rowe C , Weisler M . 2007 Berberass: marine resource specialisation and environmental change in Torres Strait during the past 4000 years. Archaeol. Oceania. **42** , 49–64. (10.1002/j.1834-4453.2007.tb00016.x)

[33] Findlay KP , Cockcroft VG , Guissamulo AT . 2011 Dugong abundance and distribution in the Bazaruto Archipelago, Mozambique. Afr. J. Mar. Sci. **33** , 441–452. (10.2989/1814232X.2011.637347)

[34] Pusineri C , Kiszka J , Quillard M , Caceres S . 2013 The endangered status of dugongs Dugong dugon around Mayotte (East Africa, Mozambique Channel) assessed through interview surveys. Afr. J. Mar. Sci. **35** , 111–116. (10.2989/1814232X.2013.783234)

[35] Hanafy M , Gheny MA , Rouphael AB , Salam A , Fouda M . 2006 The dugong, Dugong dugon, in Egyptian waters: distribution, relative abundance and threats. Zool. Middle East. **39** , 17–24. (10.1080/09397140.2006.10638178)

[36] Al-Abdulrazzak D , Pauly D . 2017 Reconstructing historical baselines for the Persian/Arabian Gulf dugong, Dugong dugon (Mammalia: Sirena). Zool. Middle East. **63** , 95–102. (10.1080/09397140.2017.1315853)

[37] Marshall CD , Al Ansi M , Dupont J , Warren C , Al Shaikh I , Cullen J . 2018 Large dugong (Dugong dugon) aggregations persist in coastal Qatar. Mar. Mammal Sci. **34** , 1154–1163. (10.1111/mms.12497)

[38] Hines EM , Adulyanukosol K , Duffus DA . 2005 Dugong (Dugong dugon) abundance along the Andaman coast of Thailand. Mar. Mammal Sci. **21** , 536–549. (10.1111/j.1748-7692.2005.tb01247.x) 16206026

[39] Meager JJ , Limpus CJ , Sumpton WD . 2013 A review of the population dynamics of dugongs in southern queensland: 1830-2012. Dept. of Environ. and Heritage Prot. 1–29.

[40] Lin M *et al* . 2022 Functional extinction of dugongs in China. R. Soc. Open Sci. **9** , 211994. (10.1098/rsos.211994) 36016916 PMC9399689

[41] Kayanne H , Hara T , Arai N , Yamano H , Matsuda H . 2022 Trajectory to local extinction of an isolated dugong population near Okinawa Island, Japan. Sci. Rep. **12** , 6151. (10.1038/s41598-022-09992-2) 35413971 PMC9005736

[42] Trebilco R , Fischer M , Hunter C , Hobday A , Thomas L , Evans K . 2021 Australia state of the environment 2021: marine, independent report to the Australian government minister for the environment. Canberra, Australia: Commonwealth of Australia. See https://soe.dcceew.gov.au/sites/default/files/2022-07/soe2021-marine.pdf.

[43] Garrigue C , Bonneville CD , Cleguer C , Oremus M . 2022 Extremely low mtDNA diversity and high genetic differentiation reveal the precarious genetic status of dugongs in New Caledonia, South Pacific. J. Hered. **113** , 516–524. (10.1093/jhered/esac029) 35665813

[44] Deutsch CJ , Castelblanco-Martínez DN , Groom R , Cleguer C . 2022 Movement behavior of manatees and dugongs: I. Environmental challenges drive diversity in migratory patterns and other large-scale movements. In Ethology and behavioral ecology of Sirenia (ed. H Marsh ), pp. 155–231. Cham, Switzerland: Springer International Publishing.

[45] McGowan AM , Lanyon JM , Clark N , Blair D , Marsh H , Wolanski E , Seddon JM . 2023 Cryptic marine barriers to gene flow in a vulnerable coastal species, the dugong (Dugong dugon). Mar. Mammal Sci. **39** , 918–939. (10.1111/mms.13021)

[46] Poommouang A *et al* . 2021 Genetic diversity in a unique population of dugong (Dugong dugon) along the sea coasts of Thailand. Sci. Rep. **11** , 11624. (10.1038/s41598-021-90947-4) 34078973 PMC8172547

[47] Marsh H , O’Shea TJ , Reynolds III JE . 2011 Ecology and conservation of the Sirenia: dugongs and manatees. Cambridge, UK: Cambridge University Press.

[48] Baker DN *et al* . 2024 A chromosome-level genome assembly for the dugong (Dugong dugon). J. Hered. **115** , 212–220. (10.1093/jhered/esae003) 38245832 PMC10936554

[49] Hofreiter M , Paijmans JLA , Goodchild H , Speller CF , Barlow A , Fortes GG , Thomas JA , Ludwig A , Collins MJ . 2015 The future of ancient DNA: technical advances and conceptual shifts. Bioessays **37** , 284–293. (10.1002/bies.201400160) 25413709

[50] Allentoft ME , Heller R , Oskam CL , Lorenzen ED , Hale ML , Gilbert MTP , Jacomb C , Holdaway RN , Bunce M . 2014 Extinct New Zealand megafauna were not in decline before human colonization. Proc. Natl Acad. Sci. USA **111** , 4922–4927. (10.1073/pnas.1314972111) 24639531 PMC3977255

[51] Star B , Barrett JH , Gondek AT , Boessenkool S . 2018 Ancient DNA reveals the chronology of walrus ivory trade from Norse Greenland. Proc. R. Soc. B **285** , 20180978. (10.1098/rspb.2018.0978) PMC6111184 30089624

[52] Star B *et al* . 2017 Ancient DNA reveals the Arctic origin of Viking Age cod from Haithabu, Germany. Proc. Natl Acad. Sci. USA **114** , 9152–9157. (10.1073/pnas.1710186114) 28784790 PMC5576834

[53] Arndt A , Van Neer W , Hellemans B , Robben J , Volckaert F , Waelkens M . 2003 Roman trade relationships at Sagalassos (Turkey) elucidated by ancient DNA of fish remains. J. Archaeol. Sci. **30** , 1095–1105. (10.1016/S0305-4403(02)00204-2)

[54] Oosting T , Star B , Barrett JH , Wellenreuther M , Ritchie PA , Rawlence NJ . 2019 Unlocking the potential of ancient fish DNA in the genomic era. Evol. Appl. **12** , 1513–1522. (10.1111/eva.12811) 31462911 PMC6708421

[55] Speller CF , Hauser L , Lepofsky D , Moore J , Rodrigues AT , Moss ML , McKechnie I , Yang DY . 2012 High potential for using DNA from ancient herring bones to inform modern fisheries management and conservation. PLoS One **7** , e51122. (10.1371/journal.pone.0051122) 23226474 PMC3511397

[56] Lorenzen ED *et al* . 2011 Species-specific responses of late quaternary megafauna to climate and humans. Nature **479** , 359–364. (10.1038/nature10574) 22048313 PMC4070744

[57] Ribeiro ÂM , Foote AD , Kupczok A , Frazão B , Limborg MT , Piñeiro R , Abalde S , Rocha S , da Fonseca RR . 2017 Marine genomics: news and views. Mar. Genomics **31** , 1–8. (10.1016/j.margen.2016.09.002) 27650377

[58] van der Valk T , Dalèn L . 2024 From genomic threat assessment to conservation action. Cell **187** , 1038–1041. (10.1016/j.cell.2024.01.038) 38428386

[59] Cooper A , Poinar HN . 2000 Ancient DNA: do it right or not at all. Science **289** , 1139. (10.1126/science.289.5482.1139b) 10970224

[60] Gilbert MTP , Bandelt HJ , Hofreiter M , Barnes I . 2005 Assessing ancient DNA studies. Trends Ecol. Evol. **20** , 541–544. (10.1016/j.tree.2005.07.005) 16701432

[61] Gondek AT , Boessenkool S , Star B . 2018 A stainless-steel mortar, pestle and sleeve design for the efficient fragmentation of ancient bone. BioTechniques **64** , 266–269. (10.2144/btn-2018-0008) 29939091

[62] Boessenkool S , Hanghøj K , Nistelberger HM , Der Sarkissian C , Gondek AT , Orlando L , Barrett JH , Star B . 2017 Combining bleach and mild predigestion improves ancient DNA recovery from bones. Mol. Ecol. Resour. **17** , 742–751. (10.1111/1755-0998.12623) 27790833

[63] Lord E *et al* . 2022 Population dynamics and demographic history of Eurasian collared lemmings. BMC Ecol. Evol. **22** , 126. (10.1186/s12862-022-02081-y) 36329382 PMC9632076

[64] Star B *et al* . 2014 Palindromic sequence artifacts generated during next generation sequencing library preparation from historic and ancient DNA. PLoS One **9** , e89676. (10.1371/journal.pone.0089676) 24608104 PMC3946424

[65] Kapp JD , Green RE , Shapiro B . 2021 A fast and efficient single-stranded genomic library preparation method optimized for ancient DNA. J. Hered. **112** , 241–249. (10.1093/jhered/esab012) 33768239 PMC8141684

[66] Schubert M *et al* . 2014 Characterization of ancient and modern genomes by SNP detection and phylogenomic and metagenomic analysis using PALEOMIX. Nat. Protoc. **9** , 1056–1082. (10.1038/nprot.2014.063) 24722405

[67] DNA Zoo Consortium . dnazoo.org.

[68] Foote AD *et al* . 2015 Convergent evolution of the genomes of marine mammals. Nat. Genet. **47** , 272–275. (10.1038/ng.3198) 25621460 PMC4644735

[69] Dudchenko O *et al* . 2017 De novo assembly of the Aedes aegypti genome using Hi-C yields chromosome-length scaffolds. Science **356** , 92–95. (10.1126/science.aal3327) 28336562 PMC5635820

[70] Jónsson H , Ginolhac A , Schubert M , Johnson PLF , Orlando L . 2013 mapDamage2.0: fast approximate Bayesian estimates of ancient DNA damage parameters. Bioinformatics **29** , 1682–1684. (10.1093/bioinformatics/btt193) 23613487 PMC3694634

[71] Li H *et al* . 2009 The sequence alignment/map format and SAMtools. Bioinformatics **25** , 2078–2079. (10.1093/bioinformatics/btp352) 19505943 PMC2723002

[72] Danecek P *et al* . 2011 The variant call format and VCFtools. Bioinformatics **27** , 2156–2158. (10.1093/bioinformatics/btr330) 21653522 PMC3137218

[73] Purcell S *et al* . 2007 PLINK: a tool set for whole-genome association and population-based linkage analyses. Am. J. Hum. Genet. **81** , 559–575. (10.1086/519795) 17701901 PMC1950838

[74] RStudio Team . 2020 Rstudio: integrated development for R. Boston, MA: RStudio, PBC.

[75] Wickham H *et al* . Welcome to the Tidyverse. JOSS **4** , 1686. (10.21105/joss.01686)

[76] Wickham H . 2016 ggplot2: elegant graphics for data analysis. Cham, Switzerland: Springer-Verlag.

[77] Minh BQ , Schmidt HA , Chernomor O , Schrempf D , Woodhams MD , von Haeseler A , Lanfear R . 2020 IQ-TREE 2: new models and efficient methods for phylogenetic inference in the genomic era. Mol. Biol. Evol. **37** , 1530–1534. (10.1093/molbev/msaa015) 32011700 PMC7182206

[78] Rambaut A . 2018 Figtree: GitHub repository. See https://github.com/rambaut/figtree.

[79] Matschiner M . 2016 Fitchi: haplotype genealogy graphs based on the Fitch algorithm. Bioinformatics **32** , 1250–1252. (10.1093/bioinformatics/btv717) 26656006

[80] Silva D . 2014 El concatenero: GitHub repository. See https://github.com/ODiogoSilva/ElConcatenero.

[81] Rozas J , Ferrer-Mata A , Sánchez-DelBarrio JC , Guirao-Rico S , Librado P , Ramos-Onsins SE , Sánchez-Gracia A . 2017 DnaSP 6: DNA sequence polymorphism analysis of large data sets. Mol. Biol. Evol. **34** , 3299–3302. (10.1093/molbev/msx248) 29029172

[82] Excoffier L , Lischer HEL . 2010 Arlequin suite ver 3.5: a new series of programs to perform population genetics analyses under Linux and Windows. Mol. Ecol. Resour. **10** , 564–567. (10.1111/j.1755-0998.2010.02847.x) 21565059

[83] Tajima F . 1989 Statistical method for testing the neutral mutation hypothesis by DNA polymorphism. Genetics **123** , 585–595. (10.1093/genetics/123.3.585) 2513255 PMC1203831

[84] Fu YX , Li WH . 1993 Statistical tests of neutrality of mutations. Genetics **133** , 693–709. (10.1093/genetics/133.3.693) 8454210 PMC1205353

[85] Drummond AJ , Rambaut A , Shapiro B , Pybus OG . 2005 Bayesian coalescent inference of past population dynamics from molecular sequences. Mol. Biol. Evol. **22** , 1185–1192. (10.1093/molbev/msi103) 15703244

[86] Bouckaert R *et al* . 2019 BEAST 2.5: an advanced software platform for Bayesian evolutionary analysis. PLoS Comput. Biol. **15** , e1006650. (10.1371/journal.pcbi.1006650) 30958812 PMC6472827

[87] Edgar RC . 2004 MUSCLE: multiple sequence alignment with high accuracy and high throughput. Nucleic Acids Res. **32** , 1792–1797. (10.1093/nar/gkh340) 15034147 PMC390337

[88] Tamura K , Stecher G , Kumar S . 2021 Mega11: molecular evolutionary genetics analysis version 11. Mol. Biol. Evol. **38** , 3022–3027. (10.1093/molbev/msab120) 33892491 PMC8233496

[89] Darriba D . 2016 jmodeltest2: GitHub repository. See https://github.com/ddarriba/jmodeltest2.

[90] Rambaut A , Drummond AJ , Xie D , Baele G , Suchard MA . 2018 Posterior summarization in Bayesian phylogenetics using tracer 1.7. Syst. Biol. **67** , 901–904. (10.1093/sysbio/syy032) 29718447 PMC6101584

[91] Tajima F . 1989 The effect of change in population size on DNA polymorphism. Genetics **123** , 597–601. (10.1093/genetics/123.3.597) 2599369 PMC1203832

[92] Gole S , Prajapati S , Prabakaran N , Johnson JA , Sivakumar K . 2023 Herd size dynamics and observations on the natural history of dugongs (Dugong dugon) in the Andaman Islands, India. Aquat. Mamm. **49** , 53–61. (10.1578/AM.49.1.2023.53)

[93] Kim HL *et al* . 2023 Prehistoric human migration between Sundaland and South Asia was driven by sea-level rise. Commun. Biol. **6** , 150. (10.1038/s42003-023-04510-0) 36739308 PMC9899273

[94] Dussex N , von Seth J , Robertson BC , Dalén L . 2018 Full mitogenomes in the critically endangered Kākāpō reveal major post-glacial and anthropogenic effects on neutral genetic diversity. Genes **9** , 220. (10.3390/genes9040220) 29671759 PMC5924562

[95] Robin M , Ferrari G , Akgül G , Münger X , von Seth J , Schuenemann VJ , Dalén L , Grossen C . 2022 Ancient mitochondrial and modern whole genomes unravel massive genetic diversity loss during near extinction of Alpine ibex. Mol. Ecol. **31** , 3548–3565. (10.1111/mec.16503) 35560856 PMC9328357

[96] Heller R , Chikhi L , Siegismund HR . 2013 The confounding effect of population structure on Bayesian skyline plot inferences of demographic history. PLoS One **8** , e62992. (10.1371/journal.pone.0062992) 23667558 PMC3646956

[97] Blair D , McMahon A , McDonald B , Tikel D , Waycott M , Marsh H . 2014 Pleistocene sea level fluctuations and the phylogeography of the dugong in Australian waters. Mar. Mammal Sci. **30** , 104–121. (10.1111/mms.12022)

[98] Furness LH , Kersten O , Boilard A , Keith Diagne K , Cristina L , Harold J . 2024 Data from: population structure of dugong dugon across the indo-pacific revealed by historical mitogenomes. Figshare. (10.6084/m9.figshare.c.7370693) PMC11304337 39113775

